# Notes

**Published:** 1899-07

**Authors:** 


### NOTES.

The infant mortality in the boroughs of Manhattan and Bronx, of Greater New York, is said to have been lessened 15 per cent. in the past five years, largely attributable to an improved milk-supply.

Dr. Tennant, of London, Ontario, reports a series of cases of parturient apoplexy successfully treated with the iodide of potassium.

Graduates in veterinary medicine are admitted to the Medical Department of the Medico-Chirurgical College, Philadelphia, on the same basis as graduates in dentistry and pharmacy.

Dr. Hoskins records two very pronounced cases of azoturia in July, when the weather was unusually hot. Both heavy, lymphatic types of draught-horses, with preceding periods of idleness of two and five days, respectively; both heavy in flesh, and complete paraplegia for a number of hours in each, with very dark, characteristically-colored urine, but in both cases a complete absence of the board-like condition of the gluteal muscles, this region, on the contrary, being flaccid. This would point strongly to the fact that cold must have some strong influence in contributing to the spasmodic contraction of these muscles as usually noted in the colder seasons.

The State Department, in replying to a criticism from Norway as to uninspected horse-meat shipped there from the United States, has suggested to the Norwegian authorities that they demand said inspection through their representatives, and the United States Government will inaugurate the same.

The Veterinary Congress, at Baden-Baden in August, will have among the attractions some three excursions among the public slaughter-houses, cattle-yards, and to investigate the system of cattle insurance maintained in certain parts of Germany.

The Chicago Veterinary Society has appointed a committee on tuberculosis who will endeavor to secure the enactment of a city ordinance requiring some form of veterinary inspection of the dairies furnishing the city with milk. The members of the committee are: Drs. Frank Allen, O. E. Dyson, L. A. Merillat, R. G. Walker, and Joseph Hughes.

The truth that prevails in a horse-trade is, indeed, mighty scarce.

Some eighteen applicants for license were before the Pennsylvania State Board of Veterinary Medical Examiners in June.

Mr. Hickox, of the graduating class of the Grand Rapids Veterinary College, in his address, after speaking of the close of their student days, referred to the wonderful progress in veterinary science during the nineteenth century; the entire development of collegiate opportunities during the last half of the century; their slow growth and meagre support in their earlier years, at times making the ultimate success of these projects doubtful, hence the sinking out of notice of the schools at Boston and Philadelphia, and the precarious existence of the school at Toronto in the period of years prior to 1875. Touching upon the development of the Grand Rapids Veterinary College in two years, he said that there were twelve students the opening year and fourteen the second; he compared the clinical advantages now offered by the newer colleges with the absence of all clinical instruction in earlier years; the faithful work of the faculty of the Grand Rapids Veterinary College was feelingly and appreciatively referred to, closing with an appeal to his fellow-graduates for steadfastness in purpose and zeal in their work, with confidence that success would follow.

